# Inverse Association Between Regional Availability of Board‐Certified Fellows of the Japan Gastroenterological Endoscopy Society and Colorectal Cancer Mortality in Japan: An Ecological Analysis

**DOI:** 10.1002/jgh3.70477

**Published:** 2026-10-01

**Authors:** Kazuya Inoki, Mahbubur Rahman, Sayaka Mizuno, Norihiro Suzuki, Shinya Nakatani, Kazuo Kikuchi, Atsushi Katagiri, Hitoshi Yoshida, Naomi Kakushima

**Affiliations:** ^1^ Division of Gastroenterology, Department of Medicine Showa Medical University School of Medicine Tokyo Japan; ^2^ Graduate School of Public Health, St. Luke's International University Tokyo Japan

**Keywords:** colorectal neoplasms, endoscopy, mortality, risk factors, social determinants of health

## Abstract

**Aims:**

We investigated the association between prefecture‐level colorectal cancer (CRC) mortality and contributing factors, including the regional availability of Board‐Certified Fellows of the Japan Gastroenterological Endoscopy Society (BCFJGES), using publicly available data from all 47 Japanese prefectures.

**Methods and Results:**

This prefecture‐level ecological cross‐sectional study used publicly available national data from e‐Stat, Cancer Statistics by the Japan National Cancer Center, and JGES records. The dependent variable was the CRC‐specific age‐standardized mortality rate (AMR) among individuals aged < 75 years in 2021. Independent variables (2010–2016) included CRC screening and follow‐up rates, smoking, alcohol consumption, obesity, physical activity, diabetes prevalence, red meat consumption, mean income, gastroenterologist and BCFJGES density per 10 000 population, CRC stage distribution, and regional classification. Simple linear regression showed that smoking, alcohol consumption, and obesity were significantly associated with higher CRC AMR, whereas physical activity, income, and BCFJGES density were associated with lower AMR. Multiple linear regression confirmed independent associations for smoking (regression coefficient [*b*] = 0.213, *p* = 0.011), alcohol consumption (*b* = 0.045, *p* = 0.003), physical activity (*b* = −0.087, *p* = 0.024), and BCFJGES density (*b* = −1.339, *p* < 0.001). The Pearson correlation coefficient between AMR and BCFJGES density was −0.482 (*p* < 0.001).

**Conclusion:**

Higher prefecture‐level smoking and alcohol consumption were associated with higher CRC mortality, while higher physical activity and higher BCFJGES density were associated with lower mortality. Regional disparities in BCFJGES distribution across Japan may contribute to CRC mortality and warrant continued surveillance and policy attention.

## 1 Introduction

Colorectal cancer (CRC) screening with fecal occult blood tests (FOBT) and subsequent colonoscopy (CS) reduces CRC mortality, as shown in several large‐scale randomized controlled trials [1, 2, 3, 4]. A fecal immunochemical test (FIT) was subsequently developed to overcome FOBT limitations, and its effectiveness has been demonstrated in Japanese settings [5, 6, 7]. An organized, population‐based CRC screening program using FOBT/FIT has been implemented in Japan since 1992. A recent US analysis showed that approximately 80% of the recent reduction in CRC mortality is attributable to screening rather than to advances in treatment [8]. Nevertheless, the corresponding reduction in CRC mortality in Japan has been less pronounced, suggesting differences in screening uptake, implementation quality, or population‐level risk factors.

Key quality indicators (QIs) for organized CRC screening include the CRC screening rate (SR) and the detailed examination rate—the proportion of FIT‐positive participants who undergo follow‐up CS—both of which directly affect screening effectiveness [9, 10, 11, 12]. Established risk factors for CRC incidence in Japan include family history [13], smoking [14], alcohol consumption [15], obesity [16], low physical activity [17], Type 2 diabetes mellitus (T2DM) [18], and red meat intake [19]. CS quality, reflected in the adenoma detection rate, is another determinant of CRC mortality. The Japan Gastroenterological Endoscopy Society (JGES) is Japan's national professional body for gastrointestinal endoscopy and administers a board certification system to maintain the quality of endoscopic practice among certified specialists. We therefore hypothesized that the regional distribution of Board‐Certified Fellows of the JGES (BCFJGES) may be an important determinant of CRC mortality in Japan.

Although the QIs of CRC screening programs and risk factors vary by prefecture, their impact on Japanese regional CRC mortality remains uncertain. Widening health disparities across prefectures have been reported in Japan [20]; however, their effect on CRC mortality has not been fully studied. This study aimed to examine the association between geographic variation in CRC risk factors—including the prefecture‐level density of BCFJGES—and prefecture‐level CRC mortality in Japan using publicly available data.

## 2 Methods

### 2.1 Study Design and Data Collection

We collected prefecture‐level data on the CRC‐specific AMR among individuals aged < 75 years, CRC screening QIs, and CRC risk factors from publicly available national databases. The QIs included screening and detailed examination rates. Risk factors comprised smoking, alcohol consumption, obesity, physical activity, T2DM, and red meat consumption, which are categorized as probable or convincing CRC risk factors in Japan by the National Cancer Center Institute for Cancer Control. The BMI ≥ 25 cutoff for obesity follows the Asia‐Pacific WHO definition [21]. Other variables included mean income, prefecture‐level CRC stage distribution, the total number of gastroenterologists, and the number of BCFJGES; the latter two were examined separately to distinguish board‐certified endoscopic capacity from overall gastrointestinal specialist supply.

We classified Japanese prefectures into eight regions. Hokkaido was included in the Hokkaido region. Aomori, Iwate, Miyagi, Akita, Yamagata, and Fukushima were included in the Tohoku region. Ibaraki, Tochigi, Gunma, Saitama, Chiba, Tokyo, and Kanagawa were included in the Kanto region. Niigata, Toyama, Ishikawa, Fukui, Yamanashi, Nagano, Gifu, Shizuoka, and Aichi were included in the Chubu region. Mie, Shiga, Kyoto, Osaka, Hyogo, Nara, and Wakayama were included in the Kinki region. Tottori, Shimane, Okayama, Hiroshima, and Yamaguchi were included in the Chugoku region. Tokushima, Kagawa, Ehime, and Kochi were included in the Shikoku region, and Fukuoka, Saga, Nagasaki, Kumamoto, Oita, Miyazaki, Kagoshima, and Okinawa were included in the Kyushu region.

### 2.2 Data Sources

#### 2.2.1 AMR

The prefecture‐wise AMR data represent CRC‐specific mortality rates and were obtained from Cancer Statistics, Cancer Information Service, and the National Cancer Center, Japan. AMR was calculated for individuals aged < 75 years and was directly standardized using the 1985 Japanese model population as the reference standard (https://ganjoho.jp/reg_stat/statistics/data/dl/en.html).

#### 2.2.2 Screening and Detailed Examination Rates

The CRC screening and detailed examination uptake rates were obtained from compilations released by the Cancer Registry and Statistics, Cancer Information Service, and National Cancer Center, Japan (https://ganjoho.jp/reg_stat/statistics/stat/screening/dl_screening.html). The two indicators are derived from different primary data sources. The SR is taken from the Comprehensive Survey of Living Conditions (Kokumin Seikatsu Kiso Chōsa) conducted by the Ministry of Health, Labour and Welfare, a nationally representative household survey based on self‐reported participation in CRC screening (including both municipal population‐based screening and workplace‐based opportunistic screening) within the past 1 year among respondents aged ≥ 40 years; the denominator is the surveyed population in each prefecture, and recall and reporting bias are recognized limitations. The detailed examination (follow‐up CS) rate, in contrast, is derived from the Report on Regional Public Health Services and Health Promotion Services, which captures the actual proportion of FIT‐positive individuals who underwent a follow‐up CS within municipal population‐based screening programs and does not include workplace‐based screening.

#### 2.2.3 Smoking Rate

The prefecture‐wise smoking rate was obtained from the Cancer Registry and Statistics, Cancer Information Service, and National Cancer Center, Japan (https://ganjoho.jp/reg_stat/statistics/data/dl/en.html).

#### 2.2.4 Alcohol Consumption

The prefecture‐wise alcohol consumption data were obtained from the National Tax Agency (https://www.nta.go.jp/publication/statistics/kokuzeicho/tokei.htm) and represent the prefectural sales volume of alcoholic beverages. The value was divided by the prefectural population and the population‐normalized figure was used for the analysis.

#### 2.2.5 Obesity Prevalence

Obesity prevalence data were obtained from the 2010 National Health and Nutrition Survey (NHNS) conducted by the Ministry of Health, Labour and Welfare, Japan, and accessed via e‐Stat (https://www.e‐stat.go.jp/stat‐search/database?statdisp_id=0003234776). The 2010 NHNS included an expanded prefecture‐level sample (approximately 200 000 individuals nationwide drawn from designated survey districts) and is the most recent NHNS round that allows prefecture‐level estimation of obesity prevalence at the time of analysis. Obesity was defined as a body mass index ≥ 25 kg/m^2^, following the Asia‐Pacific WHO definition [21]. Because the NHNS samples a subset of the residents of each prefecture rather than the entire population, the estimates carry some sampling uncertainty.

#### 2.2.6 Physical Activity

Physical activity data were obtained from prefectural statistics listed in e‐Stat (https://www.e‐stat.go.jp/regional‐statistics/ssdsview/prefectures), which are based on the Survey on Time Use and Leisure Activities (Shakai Seikatsu Kihon Chōsa) conducted by the Statistics Bureau of Japan. These data represent the proportion of residents who performed exercise for more than 10 min per session at least once per week in each prefecture.

#### 2.2.7 T2DM Status

The prefecture‐wise T2DM data were obtained from the System of Social and Demographic Statistics of Japan listed in e‐Stat (https://www.e‐stat.go.jp/regional‐statistics/ssdsview/prefectures), which is based on the Patient Survey (Kanja Chōsa) conducted by the Ministry of Health, Labour and Welfare every 3 years; the 2014 dataset (the closest year to 2016) was used. T2DM data represented the estimated number of patients with diabetes mellitus visiting outpatient clinics. Since > 90% of patients with diabetes mellitus in Japan have T2DM [22], these patient counts were assumed to represent T2DM. The number of cases was divided by the prefectural population, and the prefectural population‐normalized value was used for the analysis.

#### 2.2.8 Red Meat Consumption

The prefecture‐wise red meat consumption data were obtained from the Family Income and Expenditure Survey (Kakei Chōsa) on e‐Stat (https://www.e‐stat.go.jp/stat‐search/files?tclass=000000330003&cycle=7&year=20220), which reports the amount of household expenditure on red meat in two‐or‐more‐person households of each prefectural capital. The amount of funds used to purchase red meat was divided by the survey population, and the resulting per‐person value was used for the analysis.

#### 2.2.9 Income

The prefecture‐wise income data were obtained from the basic survey on wage structure conducted by the Ministry of Health, Labour and Welfare (https://www.e‐stat.go.jp/stat‐search/files?stat_infid=000040029286). The value of income represents the mean wage in June.

#### 2.2.10 Gastroenterologists and BCFJGES

The number of gastroenterologists per prefecture was computed from the Survey of Physicians, Dentists, and Pharmacists listed in e‐Stat (https://www.e‐stat.go.jp/stat‐search/files?tclass=000001097738&cycle=7&year=20160), which counts physicians whose primary self‐reported specialty is gastroenterology. This count was divided by the prefectural population and used as the prefecture‐level gastroenterologist density per 10 000 population. The number of BCFJGES was obtained directly from the JGES in response to a formal inquiry; this value was divided by the prefectural population and used as the prefecture‐level BCFJGES density per 10 000 population. JGES board certification requires candidates to have at least 5 years of postgraduate clinical experience, documented performance of a minimum of 1000 upper gastrointestinal endoscopic procedures and 300 colonoscopies at accredited training institutions, successful completion of the certification examination, and renewal every 5 years through continuing medical education and re‐credentialing. Because BCFJGES status requires both substantial CS experience and renewed credentialing, this variable was treated as a proxy for the regional certified endoscopic capacity relevant to CS in clinical practice. Although passive license‐holders (i.e., BCFJGES who do not currently perform CS) cannot be fully excluded, the 5‐year renewal requirement minimizes this concern. The total gastroenterologist count and BCFJGES count are therefore conceptually distinct: the former reflects the broad supply of gastroenterology specialists, including those in hepatology, gastroenterological surgery, and other fields less directly related to endoscopy, whereas the latter captures certified endoscopic capacity for CS‐based CRC detection.

#### 2.2.11 Stage of CRC

The CRC stage was calculated based on data from the Cancer Information Service, the National Cancer Center, Japan, and the Annual Report of Hospital‐Based Cancer Registries (https://jhcr‐cs.ganjoho.jp/hbcrtables/).

#### 2.2.12 Data Years

Because CRC screening has more robust evidence for reducing CRC mortality than other risk factors, the analytic dataset was anchored on the SR. Based on prior studies of organized screening, we assumed that approximately 5 years would be required for variations in upstream variables to manifest in mortality [23, 24]. Accordingly, exposures were set to 2016 and the AMR outcome to 2021; when 2016 data were not available, the closest year was used. The data years for each variable were AMR (2021), SR (2016), detailed examination rate (2016), smoking rate (2016), alcohol consumption (2016), obesity (2010), physical activity (2016), diabetes prevalence (2014), red meat consumption (2016), gastroenterologists (2016), BCFJGES (2016), and CRC stage distribution (2016). The spread of survey years (2010–2016) introduces temporal heterogeneity, most notably for obesity and diabetes; these were retained because they remain the most recent nationwide prefecture‐level estimates and because prefecture‐level distributions of such indicators tend to be temporally stable, but the heterogeneity is acknowledged as a limitation.

### 2.3 Statistical Analysis

All statistical analyses were performed using R software (version 4.3.0). Simple and multiple linear regression analyses were conducted with the AMR as the dependent variable and prefecture‐level characteristics as independent variables. Variables showing statistically significant associations with AMR in simple linear regression analyses were considered as candidate covariates for multivariable modeling, provided that their inclusion was also supported by findings from previous studies. Given the limited number of ecological units (*n* = 47) and the potential for multicollinearity among candidate covariates, the final multivariable model was restricted to four independent variables selected on the basis of epidemiological relevance and intervariable correlations. As an exploratory assessment of model specification, stepwise variable selection identified the same set of covariates as that included in the primary multivariable model. Bivariate associations were additionally assessed using Pearson and Spearman rank correlation coefficients.

To further evaluate the robustness of the multivariable regression results to individual prefectures, a leave‐one‐prefecture‐out analysis was performed. The multivariable model was refitted iteratively after excluding each of the 47 prefectures in turn, and the regression coefficient for BCFJGES was recorded for each iteration. Robustness was assessed by examining the range and direction of these estimates relative to the coefficient obtained from the full model. All *p*‐values were two‐sided, and *p* < 0.05 was considered statistically significant.

## 3 Results

### 3.1 Japanese Regional Characteristics Regarding AMR and Risk Factors for CRC

The Japanese regional characteristics data regarding AMR and CRC risk factors showed heterogeneity for CRC incidence, AMR, and other risk factors across regions (Table 1). A numerically higher CRC AMR was observed in Hokkaido and the Tohoku region. Only these two regions displayed two‐digit AMR values, and the difference between the Kinki (where AMR was lowest) and other regions was more than 2 points.

**TABLE 1:** Prefecture‐level data on age‐standardized colorectal cancer mortality (AMR 2021) and related risk factors in Japan.

	Japanese regions							
	Hokkaido	Tohoku	Kanto	Chubu	Kinki	Chugoku	Shikoku	Kyushu
AMR, mean ± SD (/10^5^)	11.20	11.00 ± 1.99	9.74 ± 0.26	9.17 ± 0.76	8.81 ± 0.74	9.59 ± 0.96	8.83 ± 0.56	9.81 ± 1.51
SR, mean ± SD (%)	34.14	46.65 ± 4.31	40.60 ± 1.58	41.33 ± 3.37	35.91 ± 2.53	38.82 ± 4.98	37.18 ± 6.03	36.26 ± 2.16
Detailed examination rate, mean ± SD (%)	66.39	79.53 ± 4.57	65.54 ± 7.78	73.84 ± 4.84	70.33 ± 8.41	72.42 ± 4.00	77.85 ± 4.42	74.62 ± 6.97
Smoking, mean ± SD (%)	24.70	21.57 ± 1.66	20.80 ± 1.29	19.49 ± 0.81	18.39 ± 0.99	18.44 ± 0.48	18.02 ± 0.90	19.39 ± 1.44
Alcohol, mean ± SD (kL/year/10^3^ people)	73.72	72.62 ± 5.18	64.34 ± 14.54	63.58 ± 7.53	61.49 ± 10.23	65.35 ± 5.18	66.82 ± 10.58	68.50 ± 7.53
Obesity, mean ± SD (%)	38.51	36.19 ± 4.67	32.11 ± 3.74	27.20 ± 2.38	29.94 ± 5.55	28.28 ± 2.36	29.52 ± 7.07	36.50 ± 5.75
PA, mean ± SD (%)	64.90	61.38 ± 3.42	71.17 ± 2.72	67.52 ± 2.45	68.30 ± 2.68	65.56 ± 1.31	65.22 ± 1.78	66.03 ± 1.76
DM, mean ± SD (%)	1.91	1.85 ± 0.19	1.60 ± 0.25	1.78 ± 0.23	1.94 ± 0.39	1.96 ± 0.17	2.09 ± 0.20	2.09 ± 0.49
Red meat, mean ± SD (1000 yen/person)	15.91	14.28 ± 1.04	13.84 ± 1.66	14.46 ± 1.08	12.90 ± 1.91	11.77 ± 1.44	11.64 ± 1.44	12.66 ± 1.04
Income, mean ± SD (1000 yen/month)	267.60	250.32 ± 18.08	311.96 ± 31.76	282.02 ± 14.76	299.11 ± 15.46	267.06 ± 17.55	265.40 ± 7.05	250.64 ± 13.26
Gastroenterologist (/10^4^ people)	1.34	1.20 ± 0.27	0.90 ± 0.23	0.98 ± 0.17	1.22 ± 0.24	1.21 ± 0.17	1.22 ± 0.13	1.33 ± 0.28
BCFJGES density, mean ± SD (/10^4^ people)	1.52	1.16 ± 0.15	1.25 ± 0.39	1.49 ± 0.49	1.55 ± 0.39	1.61 ± 0.21	1.68 ± 0.14	1.45 ± 0.30
Stage, mean ± SD (%)								
Stages 0–I	48.57	47.38	46.75	47.10	48.18	47.32	40.23	41.58
Stages II–IV	51.43	52.62	53.25	52.90	51.82	52.68	59.77	58.42

*Note:* Values are presented as mean ± SD across prefectures within each region. For Hokkaido, which consists of a single prefecture, SD was not calculated.

Abbreviations: AMR, age‐standardized mortality rate; DM, diabetes mellitus; PA, physical activity; SD, standard deviation; SR, screening rate.

### 3.2 Association Between Risk Factors and CRC Mortality in Japan: Analysis Using Prefecture‐Level Data (Exposures Primarily 2016, AMR 2021)

The analysis employing prefecture‐level data (exposures set primarily to 2016, with the nearest available year used where 2016 data were unavailable, and AMR set to 2021) showed that the estimated regression coefficients (b) for smoking rate (*b* = 0.406, *p* < 0.001), alcohol consumption (*b* = 0.055, *p* = 0.005), obesity (*b* = 0.101, *p* = 0.001), physical activity (*b* = −0.164, *p* < 0.001), income (*b* = −0.015, *p* = 0.024), and BCFJGES density (*b* = −1.681, *p* < 0.001) were statistically significant. The b of SR was not statistically significant (*b* = 0.024, *p* = 0.543) (Table 2). We conducted multiple linear regression analysis, selecting four independent variables to avoid overfitting, given the limited sample size (*n* = 47). Although income reached statistical significance in simple linear regression (*p* = 0.024), its *p*‐value was the largest among the significant exposures, and it was therefore not retained; obesity was removed to avoid multicollinearity with physical activity. As a result, smoking rate, alcohol consumption, physical activity, and BCFJGES density were included in the final multiple linear regression model. Consistent with this, income was also dropped by stepwise model selection, which retained the same four variables. A multiple linear regression analysis showed that all factors were significantly associated with lower or higher AMR; smoking (*b* = 0.213, *p* = 0.011), alcohol consumption (*b* = 0.045, *p* = 0.003), physical activity (*b* = −0.087, *p* = 0.024), and BCFJGES density (*b* = −1.339, *p* < 0.001). In the leave‐one‐prefecture‐out analysis, the regression coefficients remained consistently negative across all 47 iterations, ranging from −1.464 to −1.109, compared with −1.339 in the primary analysis (Figure 1).

**TABLE 2:** Results of simple and multiple linear regression analyses of factors associated with age‐standardized colorectal cancer mortality (AMR 2021).

Variables	Simple linear regression		Multiple linear regression	
	Regression coefficient (*b*) (95% CI)	*p*	Regression coefficient (*b*) (95% CI)	*p*
Screening rate	0.024 (−0.055 to 0.103)	0.543		
Regions				
Hokkaido	Ref.	Ref.		
Tohoku	−0.194 (−2.632 to 2.245)	0.873		
Kanto	−1.456 (−3.870 to 0.958)	0.230		
Chubu	−2.032 (−4.412 to 0.349)	0.092		
Kinki	−2.390 (−4.804 to 0.024)	0.052		
Chugoku	−1.613 (−4.086 to 0.861)	0.195		
Shikoku	−2.370 (−4.894 to 0.156)	0.065		
Kyushu	−1.389 (−3.784 to 1.006)	0.248		
Detailed examination rate	−0.010 (−0.062 to 0.041)	0.687		
Smoking rate	0.406 (0.224–0.587)	< 0.001	0.213 (0.052–0.375)	0.011
Alcohol consumption	0.055 (0.018–0.092)	0.005	0.045 (0.016–0.073)	0.003
Obesity	0.101 (0.041–0.160)	0.001		
Physical activity	−0.164 (−0.257 to −0.072)	< 0.001	−0.087 (−0.162 to −0.012)	0.024
Diabetes mellitus	−0.537 (−1.656 to 0.583)	0.339		
Red meat consumption	8.93 × 10^−5^ (−1.33 × 10^−4^ to 3.12 × 10^−4^)	0.424		
Income	−0.015 (−0.027 to −0.002)	0.024		
Gastroenterologist density	−0.483 (−1.922 to 0.956)	0.502		
BCFJGES density	−1.681 (−2.594 to −0.768)	< 0.001	−1.339 (−2.085 to −0.594)	< 0.001
Stage				
Stages 0–I	2.075 (−4.992 to 9.142)	0.557		
Stages II–IV	−2.554 (−9.661 to 4.553)	0.473		

Abbreviations: CI, confidence interval; Ref., reference.

**FIGURE 1:**
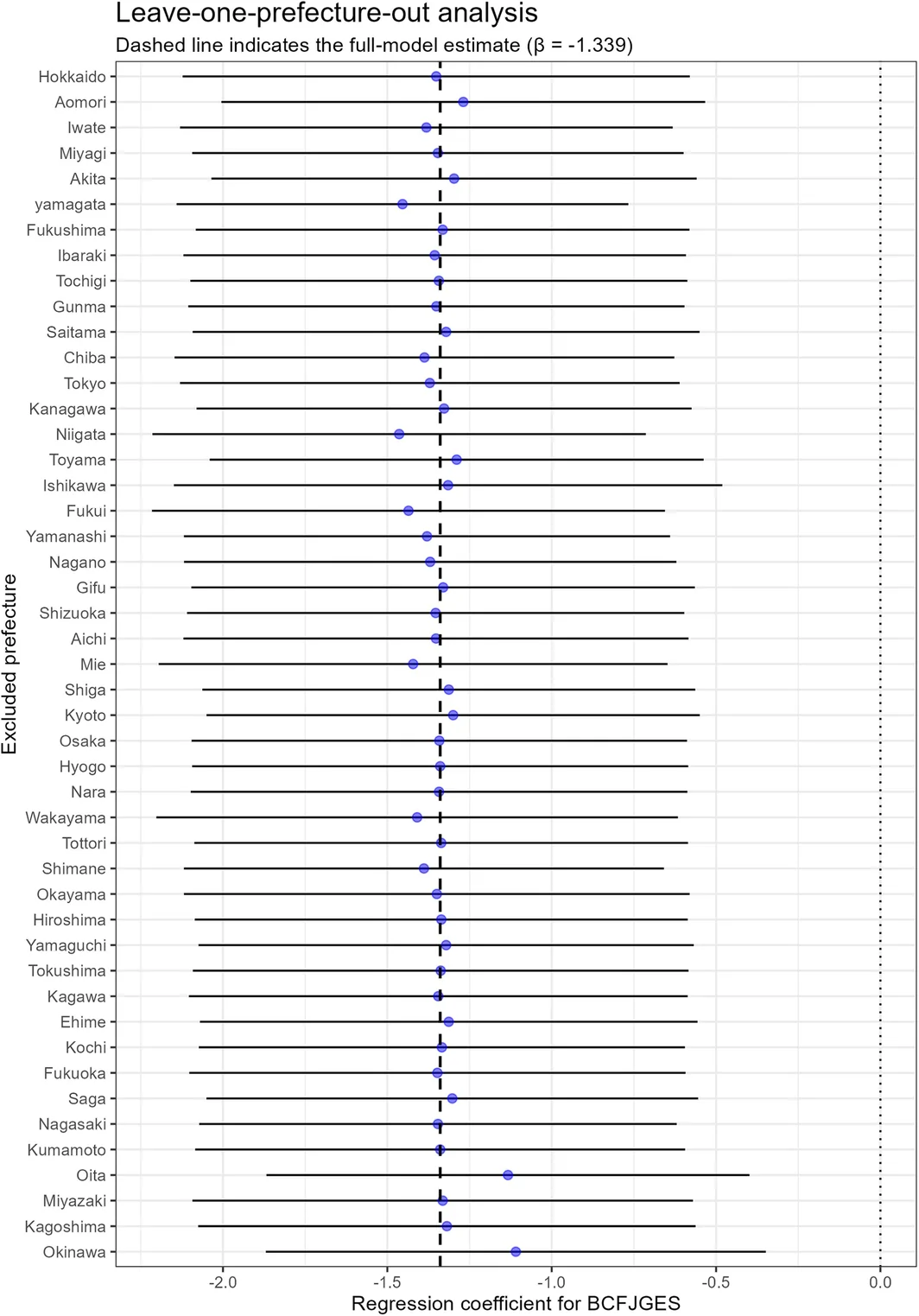
Leave‐one‐prefecture‐out sensitivity analysis. Blue circles indicate regression coefficients for BCFJGES, with horizontal lines representing 95% confidence intervals. The dashed vertical line indicates the full‐model estimate (*b* = −1.339), and the dotted vertical line indicates the null value (*b* = 0). Regression coefficients remained negative in all analyses (range, −1.464 to −1.109). *b*, regression coefficient; BCFJGES, Board‐Certified Fellows of the Japan Gastroenterological Endoscopy Society.

Figure 2 shows the correlation between AMR and the six factors that had significant association in simple linear regression. The correlation coefficient between AMR and BCFJGES density was *r* = −0.482 (*p* < 0.001). Figure 3 presents a detailed scatter plot and region‐specific regression lines illustrating a correlation between AMR and BCFJGES density by region in Japan. Four of the six prefectures in the Tohoku region are displayed on the upper left side of Figure 3 and the correlation between AMR and BCFJGES density across Japanese prefectures is displayed individually. The negative correlation between AMR and BCFJGES density has been visually preserved after regional classification.

**FIGURE 2:**
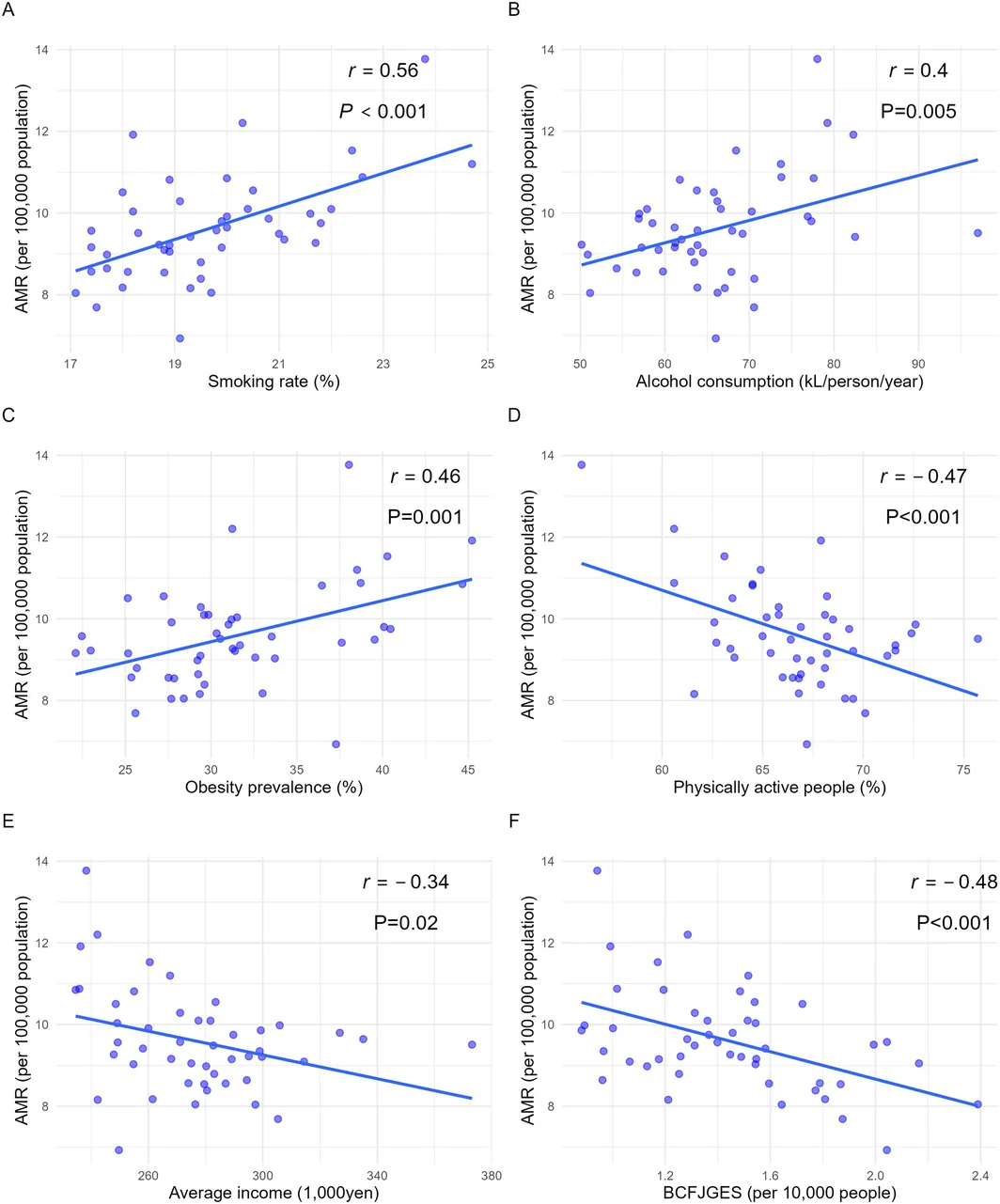
Scatterplots showing the associations between age‐standardized colorectal cancer mortality in 2021 and prefecture‐level factors significantly associated with mortality in univariable analyses. Linear regression lines and Pearson's correlation coefficients are shown. (A) AMR and smoking, (B) AMR and alcohol, (C) AMR and obesity, (D) AMR and physical activity, (E) AMR and income, and (F) AMR and BCFJGES.

**FIGURE 3:**
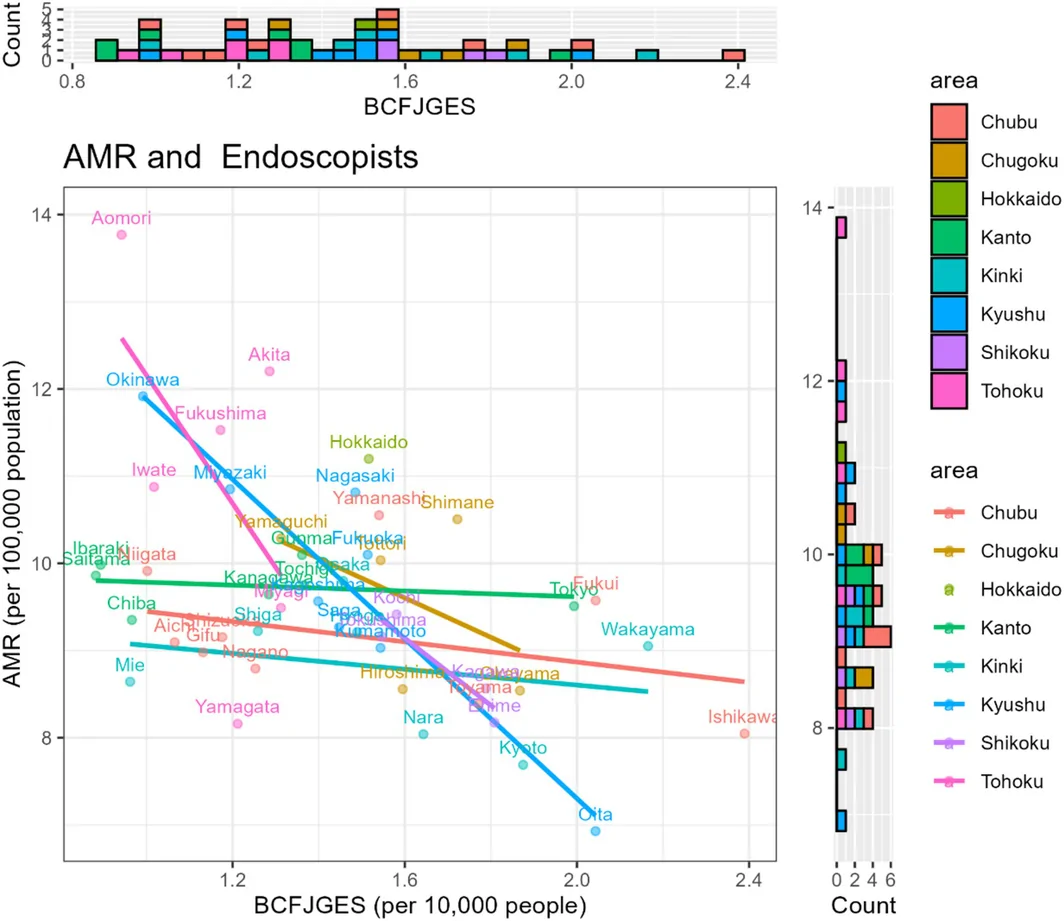
Association between age‐standardized colorectal cancer mortality in 2021 and BCFJGES density across Japanese regions. BCFJGES, Board‐Certified Fellows of the Japan Gastroenterological Endoscopy Society.

## 4 Discussion

Although the Japanese universal health insurance system ensures equitable nominal access to care, prefecture‐level disparities in health outcomes—including life expectancy and cancer mortality—have been documented [20], but the disparity for CRC has not been examined in depth. The present study revealed two key findings. First, prefecture‐level variation in CRC AMR suggests underlying social and healthcare disparities in Japan. Second, BCFJGES density, together with smoking, alcohol consumption, and physical activity, was independently associated with CRC AMR; BCFJGES density showed the largest standardized magnitude of association. Because the analysis is ecological and cross‐sectional, no individual‐level causal inference can be drawn, but the findings provide population‐level insights relevant to CRC control policy.

Significant variation in CRC AMR exists across Japanese prefectures. The CRC AMR in Aomori is approximately twice that in Oita, and elevated rates are concentrated in Tohoku and Hokkaido. The inverse association between BCFJGES density and CRC AMR is a novel and clinically plausible finding. The regression coefficient for BCFJGES density remained negative in every leave‐one‐prefecture‐out analysis, indicating that both the direction and the magnitude of this inverse association were stable to the exclusion of any single prefecture and were not driven by an individual influential prefecture. Endoscopic practice is central to CRC management through detection, diagnosis, and endoscopic resection of precancerous lesions and early CRC, and a higher adenoma detection rate is consistently associated with lower CRC incidence and mortality [25]. Several non‐mutually‐exclusive pathways are plausible: regions with more BCFJGES likely have greater CS capacity and shorter waiting times for follow‐up CS after a positive FIT; BCFJGES may achieve higher adenoma detection rates and lower post‐CS CRC rates because of standardized training and periodic re‐credentialing; and these regions may also have stronger institutional infrastructure for CRC treatment. Our data cannot distinguish among these mechanisms, but each is consistent with the direction of the observed association. Notably, BCFJGES density showed a stronger association with CRC AMR than the total number of gastroenterologists. Although the two indicators are measured from different surveys and are not strictly comparable, the result is interpretable: the gastroenterologist count is a self‐reported specialty designation that does not require demonstrated CS expertise, whereas BCFJGES certification specifically requires documented CS performance and 5‐yearly re‐credentialing. In some prefectures the count of self‐designated gastroenterologists even exceeds the count of BCFJGES, suggesting that the gastroenterologist variable is a more diffuse marker that includes non‐endoscopic clinicians (e.g., hepatologists). Endoscopic competence, rather than gastrointestinal specialist supply per se, therefore appears to be the relevant determinant; this should be confirmed using direct measures of CS volume.

Although smoking and alcohol consumption are established risk factors for CRC incidence, their role in CRC mortality is less well characterized; elevated mortality in prefectures with higher smoking and alcohol consumption may reflect not only incidence but also effects on disease progression and treatment response [26, 27]. Nicotine has been reported to induce chromosomal abnormalities [28], acetaldehyde from alcohol metabolism is genotoxic [29], and both preoperative smoking and alcohol consumption are associated with postoperative complications [30, 31].

Income was inversely associated with CRC AMR in simple linear regression but was not retained in the multivariable model and is therefore interpreted with caution. Mean prefecture‐level income may capture broader socioeconomic conditions that influence health‐seeking behavior, screening participation, and timely access to follow‐up CS and treatment, consistent with previous Japanese reports of socioeconomic disparities in cancer outcomes [32] and with recent evidence that CRC is more often diagnosed at advanced stages in socioeconomically deprived areas [33, 34]. This signal may operate through the variables retained in the multivariable model rather than as an independent determinant.

The present study was an ecological analysis using a single baseline measure of screening participation with an approximately five‐year lag before the assessment of CRC mortality. This interval may have been insufficient to capture the full mortality benefit of prolonged and repeated screening. Moreover, the SR was based on self‐reported participation in both municipal and workplace‐based screening, whereas the detailed examination rate was derived from administrative records limited to municipal programs; this difference in source populations, together with recall and reporting bias, may have attenuated prefecture‐level associations. A single baseline measure may also not adequately reflect cumulative screening exposure and did not capture repeated screening, follow‐up CS, or program quality. Therefore, the null association should not be interpreted as evidence against the effectiveness of CRC screening. This study has several limitations. First, the ecological and cross‐sectional design precludes individual‐level causal inference, although the direction of significant associations is consistent with prior individual‐level evidence. Second, exposure variables span 2010–2016, introducing temporal heterogeneity (most notably for obesity and diabetes). These variables were retained because they are the most recent prefecture‐level estimates available. Third, several exposure variables are derived from population subsamples rather than complete enumerations—including obesity and physical activity (national survey samples), red meat consumption (a survey restricted to prefectural‐capital households), and alcohol consumption (a sales‐volume proxy)—and therefore carry measurement errors. Finally, the possibility of residual confounding cannot be excluded. Several factors that could not be incorporated into our model—including urban versus rural setting, hospital case volume, the availability of colorectal surgeons, CS volume, and access to cancer treatment—may influence both the regional density of BCFJGES and CRC mortality. Further analysis is necessary to investigate these possible confounding factors.

In conclusion, in this prefecture‐level ecological analysis, higher prefecture‐level smoking and alcohol consumption were associated with higher CRC mortality, whereas higher prefecture‐level physical activity and a higher density of BCFJGES were associated with lower CRC mortality. The uneven distribution of BCFJGES across Japanese prefectures warrants continued monitoring and further investigation regarding its impact on CRC mortality, ideally through longitudinal analyses at finer spatial resolution.

## Funding

The authors have nothing to report.

## Ethics Statement

The authors have nothing to report.

## Consent

The authors have nothing to report.

## Conflicts of Interest

The authors declare no conflicts of interest.

## Data Availability

All data analyzed in this study are publicly available. Prefecture‐level CRC age‐standardized mortality, screening and detailed examination rates, and smoking rates were obtained from Cancer Statistics, Cancer Information Service, National Cancer Center, Japan (https://ganjoho.jp/reg_stat/statistics/). Alcohol sales data were obtained from the National Tax Agency (https://www.nta.go.jp/publication/statistics/). Obesity, physical activity, diabetes, and income data were obtained from e‐Stat (https://www.e‐stat.go.jp/). Red meat expenditure data were obtained from the Family Income and Expenditure Survey via e‐Stat. Numbers of gastroenterologists were obtained from the Survey of Physicians, Dentists and Pharmacists via e‐Stat. Numbers of BCFJGES were provided by the Japan Gastroenterological Endoscopy Society in response to a formal inquiry. The analytic dataset and R analysis scripts are available from the corresponding author upon reasonable request.
